# High-pressure synthesis and crystal structure of iron *sp*^3^-carbonate (Fe_2_[C_4_O_10_]) featuring pyramidal [C_4_O_10_]^4-^ anions

**DOI:** 10.1038/s42004-025-01450-0

**Published:** 2025-03-05

**Authors:** Valentin Kovalev, Dominik Spahr, Bjoern Winkler, Lkhamsuren Bayarjargal, Lena Wedek, Alena Aslandukova, Anna Pakhomova, Gaston Garbarino, Elena Bykova

**Affiliations:** 1 Goethe University Frankfurt, Institute of Geosciences, 60438 Frankfurt am Main, Germany; 2 Bayerisches Geoinstitut, University of Bayreuth, 95440 Bayreuth, Germany; 3 Material Physics and Technology at Extreme Conditions, Laboratory of Crystallography, University of Bayreuth, 95440 Bayreuth, Germany; 4 European Synchrotron Radiation Facility, 38000 Grenoble, France

**Keywords:** Solid-state chemistry, Chemical physics

## Abstract

The behavior of iron carbonates at high pressures is relevant for geological processes occurring in Earth interiors. Here, cubic iron *sp*^3^-carbonate Fe_2_[C_4_O_10_] was synthesized in diamond anvil cell by reacting Fe_2_O_3_ and CO_2_ at 65(4) GPa and 3000(±500) K, simulating the environment of localized thermal anomalies in the mantle. The crystal structure, determined by in situ single-crystal X-ray diffraction, features pyramidal [C_4_O_10_]^4-^ anions. The experimental crystal structure corresponds to a structural model from density functional theory calculations. Experimentally determined values for zero-pressure volume *V*_0_ and bulk modulus *K*_0_ are: *V*_0_ = 1059(17) Å^3^, *K*_0_ = 160(18) GPa, The DFT-calculated Raman spectrum, modeled with zinc substituting iron, matches the experimental one, supporting the structural model’s accuracy. Fe_2_[C_4_O_10_] remained stable upon decompression down to 25 GPa, below which it amorphized. DFT calculations also reveal a spin crossover of Fe^2+^ cations at 95 GPa, which is significantly higher than in other Fe^2+^-containing carbonates.

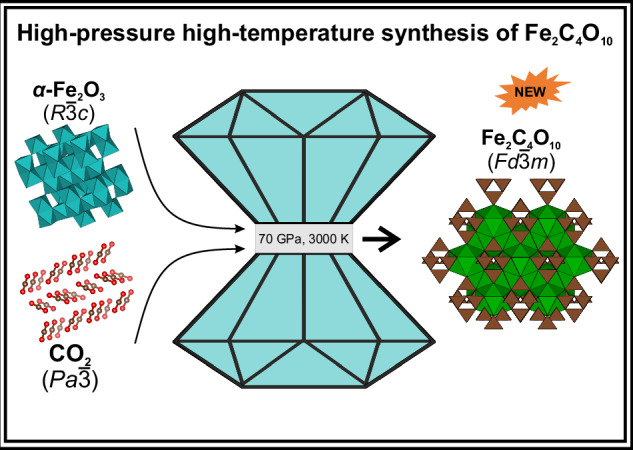

## Abstract (web summary)

Geochemically relevant [C_4_O_10_]^4-^ pyramids are the most polymerized high pressure carbonate units, with Mn-, Cd-, Ca- and Ba-based structures reported to date. Here, the authors synthesized an Fe-carbonate featuring [C_4_O_10_]^4-^ anions with a predicted high-to-low-spin crossover at unusually high pressure near 95 GPa.

## Introduction

Carbonates are widely distributed in the geological environment. They play a significant role as a carbon source in geological processes occurring in the biosphere, hydrosphere and Earth’s crust such as diagenesis, weathering and formation of ore deposits^1–5^. According to recent studies, carbonates could potentially serve as a carbon source during diamond crystallization across different geological environments, for example, as a component of carbonatites^6–10^. Moreover, carbonates are the primary constituents of marine sediments and can be transported together with water and carbonic fluids via subducting slabs into Earth’s deep interior where they are involved in redox processes on the core-mantle boundary and in the formation of deep mantle melts^3,11^.

So-called “conventional” carbonates, such as calcite and aragonite (Ca[CO_3_]), dolomite (CaMg[CO_3_]_2_), siderite (Fe[CO_3_]), and many other synthetic and natural species, contain planar trigonal [CO_3_]^2-^ anions, where the bonding orbitals of the carbon atoms show *sp*^2^-hybridisation^12,13^. Recent experiments in diamond anvil cells (DAC) confirm that carbon follows the well-known tendency to increase the coordination number with applied pressure and that it changes its coordination from *sp*^2^-trigonal to a *sp*^3^-tetrahedral coordination at pressures as low as at ~20 GPa^14–16^. It has also been shown that the tetrahedral coordination of carbon atoms can be recovered even under ambient conditions^15^. In addition, both [CO_3_]^2-^ and [CO_4_]^4-^ anions can polymerize at elevated pressures and temperatures. Such novel carbonates were obtained either by a thermal decomposition of single-source precursors^17–24^ or through a chemical reaction between conventional carbonates and CO_2_^15,16,25–28^. In case of *sp*^2^-carbon, the reactions can lead to the formation of pyrocarbonates [C_2_O_5_]^2-^ ^29–32^, while [CO_4_]^4-^ tetrahedra can be polymerized to four-membered pyramidal units [C_4_O_10_]^4-^ ^24–26^, rings [C_3_O_9_]^6-^ ^19–21,33,34^, four-membered linear groups [C_4_O_13_]^10-^ ^17,18,24^ and pyroxene-like infinite chains [C_2_O_6_]^4–23^. A few carbonates with isolated [CO_4_]^4-^ tetrahedra have been also reported^15,17,27,28^.

The rich crystal chemistry of high-pressure *sp*^3^-carbonates brings them closer to the silicates in terms of their structural diversity^35^. The degree of polymerization of the anions can be estimated using **NBO/T** (number of **n**on-**b**ridging **o**xygen per **t**etrahedron in complex anions) widely adopted for silicates^36,37^. For isolated [CO_4_]^4-^ tetrahedra the NBO/T is 4, whereas for [C_4_O_10_]^4-^ pyramids the NBO/T is 1. Thus, [C_4_O_10_]^4-^ units, having only one non-bridging oxygen atom per tetrahedron, are the most polymerized carbonate anions discovered so far. Only three anhydrous carbonates with [C_4_O_10_]^4-^ pyramids have been reported: isostructural ${{{{\rm{Mn}}}}}_{2}[{{{{\rm{C}}}}}_{4}{{{{\rm{O}}}}}_{10}]-{Fd}\bar{3}m$^24^ and ${{{{\rm{Cd}}}}}_{2}[{{{{\rm{C}}}}}_{4}{{{{\rm{O}}}}}_{10}]-{Fd}\bar{3}m$^25^ and ${{{{\rm{Ca}}}}}_{2}[{{{{\rm{C}}}}}_{4}{{{{\rm{O}}}}}_{10}]-I\bar{4}2d$^26^, whose crystal structure is derived from the cubic parent structures through a tetragonal distortion. However, the recent study of barium hydrogencarbonate with similar pyramids has demonstrated that [C_4_O_10_]^4-^ units can also be hydrated^38^. The limited amount of experimental data on the formation conditions and properties of [C_4_O_10_]^4-^ carbonates prompted us to consider the Fe-C-O system, due to the relevance of Fe-carbonates for geochemical processes such as diamond formation^39,40^ and the fact that multiple Fe-carbonates, including polymerized ones, have been already found in high-pressure experiments^17,18,20,41^.

Here we report a successful synthesis and structural characterization of a cubic iron *sp*^3^-carbonate featuring pyramidal [C_4_O_10_]^4-^ units formed in a reaction between *η*-Fe_2_O_3_, and CO_2_ at 65(4) GPa. We describe its high-pressure behavior and its stability on the decompression derived both from the experimental data and from density function theory (DFT) calculations.

## Results and discussion

### Synthesis and structural characterization of Fe_2_[C_4_O_10_]

**Fig. 1:**
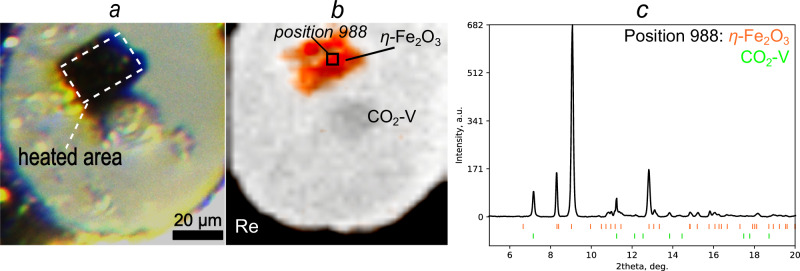
Results of the CO_2_-laser treatment of the sample. **a** Sample chamber with *η*-^57^Fe_2_O_3_ and a transparent ruby sphere in CO_2_-pressure medium after laser heating to ~2000 K at 65 GPa; (**b**) distribution of the phases based on X-ray diffraction; (**c**) XRD pattern showing the presence of *η*-Fe_2_O_3_ and CO_2_-V in the spot #988 of the XRD map.

After laser heating of Fe_2_O_3_ to ~2000 K at 65 GPa (Fig. 1a), we used XRD mapping in order to understand if a chemical reaction had occurred. We observed the appearance of new sharp reflections on the diffraction images across the heated sample. After analyzing SCXRD data collected in several points nearby the heated area, we could determine that, the peaks belong to either *η*-Fe_2_O_3_ with a post-perovskite structure (space group *Cmcm, a* = 2.6752(3) Å*, b* = 8.616(3) Å*, c* = 6.4374(14) Å) or to CO_2_-V (space group $I\bar{4}2d$, *a* = 3.4862(4) Å, *c* = 5.7085(5) Å) (Fig. 1b). Careful analysis of the heated area using powder XRD map data have shown that no other phases are present (Fig. 1c). The presence of CO_2_-V corresponds to its stability field^42^ and unit cell parameters are in an agreement with the previously determined values at similar pressures^43,44^.

According to previous studies, *η*-Fe_2_O_3_ is stable in a wide pressure range from 50 to 110 GPa at temperatures above 1500 K, while at sufficiently high temperatures (>2700–3000 K), it can partially reduce forming Fe^2+^-bearing oxides, such as Fe_5_O_7_^45^. The absence of Fe_5_O_7_ in the heated sample provides sufficient evidence that the temperature during heating did not exceed 2700 K.

**Fig. 2:**
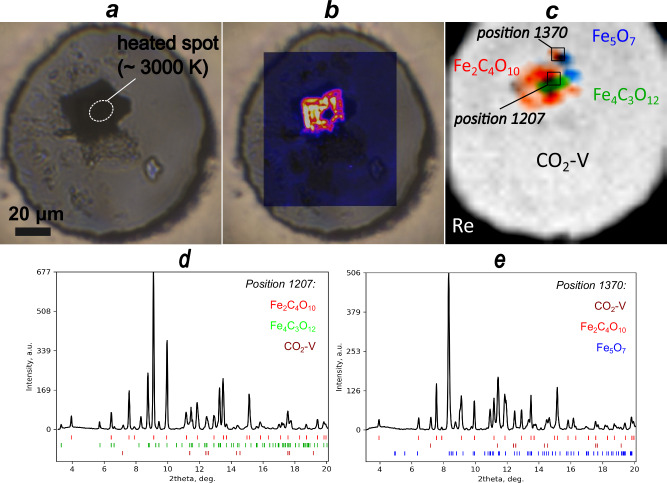
Results of YAG-laser treatment of the sample. **a** Sample chamber after heating to ~3000 K at 65 GPa localized in the central part of *η*-Fe_2_O_3_; (**b**) and (**c**)—distribution of the novel Fe_2_[C_4_O_10_] phase across the chamber based on Raman map (band wavelength 876 cm^−1^) and X-ray diffraction, respectively, together with other identified phases; XRD patterns showing identified phases in (**d**) the central part and in (**e**) the outer part of the heated sample. In the central part, the iron carbonate phase Fe^3+^_4_[C_3_O_12_] was detected, while in the outer part we observed partially reduced Fe^2+^-bearing Fe_5_O_7_, indicating different temperature conditions across the sample.

After we found that an initial heating up to ~2000 K was insufficient to induce a reaction, we performed a second laser heating experiment at higher temperatures (~3000 K) (Fig. 2a). As a result, XRD patterns measured on a 2D grid across the sample chamber changed dramatically, suggesting a reaction between the iron oxide and CO_2_ or carbon from diamond anvils. From the newly appeared reflections we could identify iron carbonate Fe^3+^_4_[C_3_O_12_] (S.G.: *R*3*c, a* = 12.9816(16) Å, *c* = 5.3019(12) Å)^17^ in the central part of the sample (Fig. 2c, d). This phase was previously observed among the products of thermal decomposition of siderite, FeCO_3_, at 74(1) GPa above 1750(100) K. In contrast, on the rims of the sides of the heated sample, we have found partially reduced Fe^2+^-bearing Fe_5_O_7_ (space group *C*2*/m*, *a* = 8.8096(7) Å, *b* = 2.6369(10) Å, *c* = 8.0464(5) Å, *β* = 105.63(8)°)^45^, suggesting that *η*-Fe_2_O_3_ experienced heating at temperatures above 2700 K^45^ (Fig. 2c, e).

**Fig. 3:**
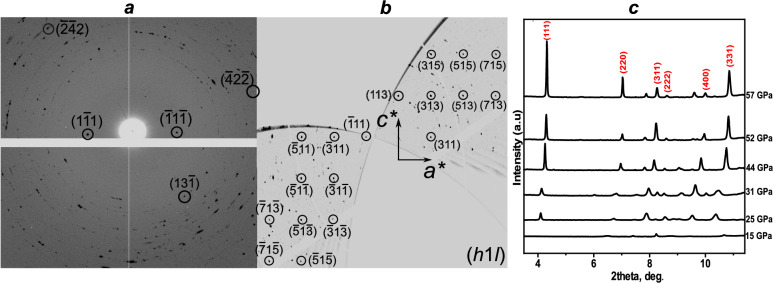
Identification of the novel phase Fe_2_[C_4_O_10_]. **a** Fragment of XRD frame at ω = 0° at 65(4) GPa with peaks belonging to Fe_2_[C_4_O_10_] (shown by black circles). White bars are gaps in DECTRIS EIGER detector; (**b**) reciprocal space reconstruction for Fe_2_[C_4_O_10_] corresponding to the (*h*1*l*) plane. Reflections belonging to Fe_2_[C_4_O_10_] are marked with black circles and located at the nodes of the unwarp grid; (**c**) XRD patterns collected for Fe_2_[C_4_O_10_] on decompression from 57 to 15 GPa demonstrating a shift of characteristic reflections to lower-2θ region and a gradual deterioration of the sample with decreasing pressure highlighted by drops in intensities of reflections and their broadening. Characteristic reflections for Fe_2_[C_4_O_10_] are shown by red.

Nevertheless, several reflections could not be assigned to Fe^3+^_4_[C_3_O_12_] or Fe_5_O_7_ (Fig. 3a). The unknown phase can be found in the whole sample volume (Fig. 2b, c). According to the analysis of our subsequently collected SCXRD data, these peaks belong to a novel iron carbonate with a chemical composition ${{{{\rm{Fe}}}}}_{2}[{{{{\rm{C}}}}}_{4}{{{{\rm{O}}}}}_{10}]-{Fd}\bar{3}m$ (*a* = 9.3992(13) Å). Based on charge balance considerations, this compound contains only Fe^2+^. We suggest that iron from the starting *η*-Fe^3+^_2_O_3_ was completely reduced after higher-temperature heating to ~3000 K at 65 GPa. The co-occurrence of Fe_2_[C_4_O_10_] with non-reduced Fe^3+^_4_C_3_O_12_ and partially reduced Fe_5_O_7_ points to the significant temperature gradients due to the non-uniform heating and insufficient thermal insulation from the diamond anvils.

The crystal structure of ${{{{\rm{Fe}}}}}_{2}[{{{{\rm{C}}}}}_{4}{{{{\rm{O}}}}}_{10}]-{Fd}\bar{3}m$ at 65 GPa was refined to *R*_1_ = 3.31%, *wR*_2_ = 9.02% with a data/parameter ratio of 126/13 (~9.6), pointing to the high quality of the refinement. For this dataset, as well as for those collected at 57, 52 and 44 GPa, the number of reflections was sufficient to refine anisotropic displacement parameters for all atoms. The resulting *R*_1_ values do not exceed 5%, with data/parameter ratio from 7.2 to 8.0, indicating the good quality of the refinements.

During decompression starting at 31 GPa, a significant reduction and broadening of the reflection intensities belonging to Fe_2_[C_4_O_10_] (Fig. 3c), can be observed, suggesting the onset of amorphization. The number of reflections also decreases drastically. To maintain a sufficient data-to-parameter ratio (6–7 reflections per refined parameter), only the iron atoms were refined using an anisotropic approximation. The last XRD dataset for Fe_2_[C_4_O_10_] with reliable structure refinement (*R*_1_ = 7.22%) was collected at 25 GPa. Below this pressure, the quality of the sample deteriorated significantly, and it was no longer possible to identify the compound from the XRD pattern (Fig. 3c). The final results of the refinements of the crystal structure of Fe_2_[C_4_O_10_] with the atomic coordinates and bond distances at different pressure points are given in Table 1.

**Table 1:** Details of crystal structure refinements for Fe_2_[C_4_O_10_] (S.G.: ${{{\rm{Fd}}}}\bar{3}{{{\rm{m}}}}$; Z = 8; Fe1 16 d (0, 0, 0.5), C1 32e (x, x, z), O1 32e (x, x, z), O2 48 f (x, 3/8, 3/8)) at high pressures

Pressure, GPa	65(4)	57(4)	52(4)	44(4)	31(4)^a^	25(4)^a^
**a, Å**	9.3392(13)	9.4330(10)	9.4862(9)	9.5696(7)	9.7291(16)	9.778(3)
**V, Å**^3^	830.4(3)	839.4(3)	853.6(2)	876.36(19)	920.9(5)	934.9(7)
**ρ**_**calc**_**, g/cm**^3^	5.115	5.060	4.976	4.847	4.612	4.543
**μ, mm**^−1^	1.194	1.517	1.492	1.453	1.383	1.362
**2Θ**_**min**_ **for data collection (°)**	3.948	4.314	4.290	4.252	6.832	4.162
**2Θ**_**max**_ **for data collection (°)**	39.086	42.318	41.990	40.404	36.756	31.140
**Reflections collected**	485	373	346	357	152	177
**Independent reflections**	126	103	94	105	68	63
**Independent reflections [I** > **2σ(I)]**	111	98	86	97	53	45
**Refined parameters**	13	13	13	13	9	9
**R**_**int**_	0.0261	0.0274	0.0357	0.0227	0.0472	0.0693
**R(σ)**	0.0260	0.0157	0.0194	0.0127	0.0500	0.0675
**R**_**1**_ **[I** > **2σ(I)]**	0.0333	0.0331	0.0304	0.0406	0.0724	0.0722
**wR**_**2**_ **[I** > **2σ(I)]**	0.0908	0.0794	0.0789	0.1203	0.1815	0.1695
**R**_**1**_	0.0379	0.0349	0.0323	0.0422	0.0883	0.0996
**wR**_**2**_	0.0917	0.0797	0.0795	0.1211	0.1899	0.1819
**S (F**^2^**)**	1.161	1.141	1.124	1.239	1.192	1.186
**Δρ**_**max**_ **(e/Å**^3^**)**	0.65	0.75	0.60	0.95	0.72	1.11
**Δρ**_**min**_ **(e/Å**^3^**)**	−0.68	−0.74	−0.67	−0.67	−0.68	−0.87
**x, z (C1)**	0.0401(3), 0.2099(3)	0.0404(2), 0.2096(2)	0.0407(2), 0.2093(2)	0.0410(4), 0.2090(4)	0.0421(12), 0.2079(12)	0.0417(14), 0.2083(14)
**x, z (O1)**	0.0378(2), 0.7122(2)	0.0376(2), 0.7124(2)	0.0372(2), 0.7128(2)	0.0367(3), 0.7133(3)	0.0344(9), 0.7156(9)	0.0349(9), 0.7151(9)
**x (O2)**	0.0435(3)	0.0433(2)	0.0428(2)	0.0416(3)	0.0385(9)	0.0376(11)
**U**_**eq**_ **(Fe1)**	0.0098(3)	0.0148(3)	0.0105(3)	0.0177(5)	0.0262(15)	0.0299(17)
**U**_**eq/iso**_ **(C1)**	0.0079(5)	0.0146(7)	0.0092(7)	0.0175(11)	0.027(4)	0.034(6)
**U**_**eq/iso**_ **(O1)**	0.0086(6)	0.0139(6)	0.0106(6)	0.0176(9)	0.028(3)	0.024(4)
**U**_**eq/iso**_ **(O2)**	0.0081(5)	0.0127(5)	0.0090(5)	0.0161(8)	0.025(2)	0.021(3)
**d (Fe1…O1), Å**	2.0567(12)	2.0656(11)	2.0790(11)	2.1005(17)	2.150(6)	2.158(6)
**d (Fe1…O2), Å**	2.555(3)	2.566(3)	2.5862(15)	2.615(3)	2.666(8)	2.710(8)
**d (C1…O1), Å**	1.269(6)	1.273(5)	1.281(5)	1.288(7)	1.29(3)	1.30(3)
**d (C1…O2), Å**	1.3750(18)	1.3778(17)	1.3810(16)	1.385(3)	1.384(8)	1.389(9)
**Data collection**	ESRF, ID27, EIGER2 X CdTe 9 M detector, λ ~ 0.37 Å	ESRF, ID15b, EIGER2 X CdTe 9 M detector, λ ~ 0.41 Å				
**CCDC number**	2393312	2393315	2393316	2393313	2393314	2393311

^a^Thermal displacements for C1, O1 and O2 are in isotropic approximation.

**Fig. 4:**
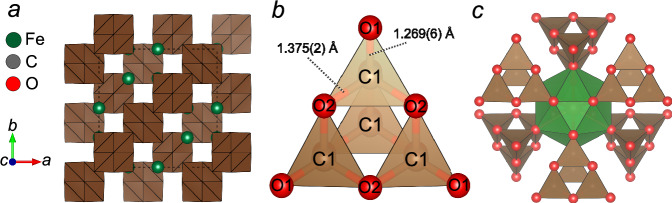
The crystal structure of Fe_2_[C_4_O_10_]. **a** View along *c*; (**b**) arrangement of [CO_4_]^4-^ tetrahedra in a polymerized pyramidal unit [C_4_O_10_]^4-^ (C-O bond lengths correspond to 65 GPa); (**c**) assemblage of [FeO_12_] icosahedra (green) and six surrounding [C_4_O_10_]^4-^ pyramids (brown) through common edges.

**Fig. 5:**
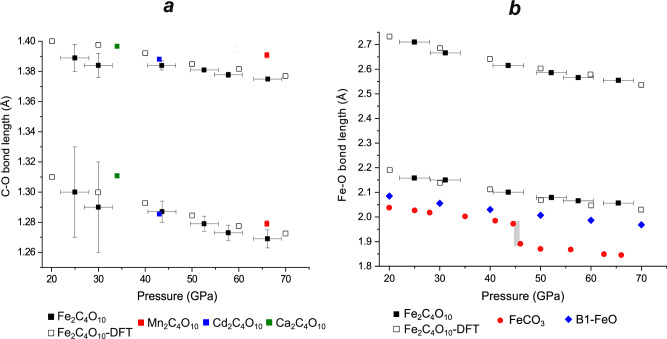
Evolution of interatomic distances with increasing pressure. **a** C-O bond lengths in high-pressure carbonates featuring [C_4_O_10_]^4-^ pyramidal units as a function of pressure; (**b**) Fe-O bond lengths in Fe_2_C_4_O_10_ and Fe^2+^-bearing compounds as a function of pressure. The error bars for some data points are smaller than symbols used.

In the crystal structure of Fe_2_[C_4_O_10_] (Fig. 4a), carbon atoms are in a *sp*^3^-hybridization state and coordinated by four oxygen atoms forming [CO_4_]^4-^ tetrahedra. Each [CO_4_]^4-^ polyhedron, in turn, shares their corners with three neighboring tetrahedra forming [C_4_O_10_]^4-^ “*super-tetrahedral*” pyramidal anions (Fig. 4b). The oxygen atoms are arranged in a close cubic packing. In the *super-tetrahedra* bridging C-O2 bonds are longer than the terminal C-O1 bonds. At 65 GPa, C-O1 and C-O2 bonds are 1.269(6) Å and 1.3750(18) Å, respectively (Table 1). Similar differences can be observed not only in the pyramidal units of isostructural carbonates Cd_2_[C_4_O_10_]^25^, Ca_2_[C_4_O_10_]^26^ and Mn_2_[C_4_O_10_]^24^ (Fig. 5a), but also in other polymerized groups, such as [C_4_O_13_]^10-^ truncated chains in Fe_4_[C_4_O_13_], [C_3_O_9_]^6-^ three-membered rings in Mg_3_[C_3_O_9_] and [C_2_O_6_]^4-^ pyroxene-like chains in high-pressure Ca[CO_3_]-*P*2_1_*/c*. At ~75 GPa, the values for terminal and bridging C-O bonds in the [C_4_O_13_]^10-^ units are reported to lie within the ranges 1.275(6)-1.340(7) Å and 1.357(9)-1.394(9) Å, respectively^17^. In the case of [C_3_O_9_]^6-^ rings in Mg_3_[C_3_O_9_] at 98 GPa, the reported bond lengths for the bridging C-O bonds are within the range 1.38(3)-1.409(19) Å, while the terminal bonds are shorter, of 1.287(18)-1.29(4) Å^46^. Similarly, for theoretically predicted Ca[CO_3_]-*P*2_1_*/c* with [C_2_O_6_]^4-^ pyroxene-like chains, terminal and bridging C-O bond lengths are reported as 1.316(0) Å and 1.415(0) Å at 60 GPa, respectively^47^.

[C_4_O_10_]^4-^ units form large cavities occupied by Fe^2+^ cations. Each Fe^2+^ cation is coordinated by twelve oxygen atoms (six Fe-O1 at a distance of 2.0567(12) Å, and six Fe-O2 at 2.555(3) Å at 65 GPa), forming a distorted icosahedron (Fig. 4c). In contrast, Fe^2+^ occupies smaller sites in structures of other Fe^2+^-bearing carbonates, such as FeCO_3_ and Fe_4_C_4_O_13_. In Fe_4_C_4_O_13_, Fe^2+^ atoms occupy bicapped prisms with a Fe-O average bond length of 2.0314 Å at 97 GPa^17^, while FeCO_3_ follows a calcite structural type, with Fe-O bond length of 1.845(3) Å at 66 GPa^48^. Based on the Fe-O bond lengths, Fe^2+^ in Fe_2_[C_4_O_10_] remains in the high-spin state up to the highest pressure studied (65 GPa). In the calculations carried out at 50, 70, and 90 GPa, both high spin and low spin configurations were obtained. In all these calculations, the enthalpies for different high spin arrangements were quite similar, where the difference between the ferromagnetic state and a high spin antiferromagnetic state was ~0.3 eV per unit cell. The low spin configurations were substantially less stable at low pressures. However, the enthalpy difference between the low-spin and high-spin states decreased from 2.65 eV per unit cell at 50 GPa linearly to 0.36 eV per unit cell at 90 GPa. An extrapolation implies that the spin crossover would be induced at 95 GPa, which is the highest known value among known Fe^2+^-bearing compounds. For example, in siderite Fe[CO_3_], having Fe^2+^ in a smaller octahedral coordination, the spin crossover occurs between 44 and 45 GPa^48–50^. This transition pressure is well reproduced by DFT-PBE-GGA calculations such as those carried out here^51^. In wüstite, B1-FeO, with an octahedral coordination of Fe^2+^ atoms, similar to that in siderite, the spin crossover occurs at 74 GPa^52,53^, but Fe-O bonds in wüstite are longer than those in siderite (Fig. 5b). Therefore, the presence of larger-volume coordination polyhedra around iron atoms can contribute to a higher pressure for spin crossover of Fe^2+^ atoms in Fe_2_[C_4_O_10_].

**Fig. 6:**
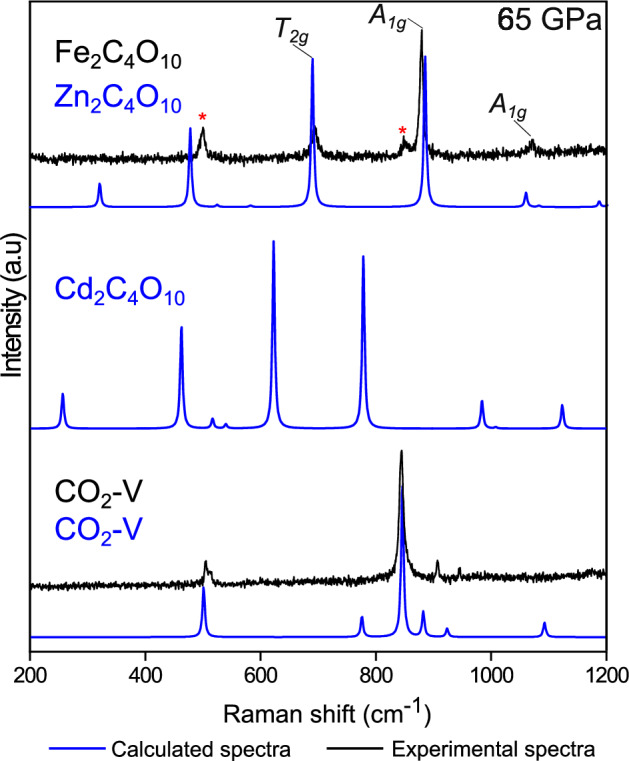
Results of spectroscopic measurements for Fe_2_[C_4_O_10_] and coexisting CO_2_-V. There is an agreement between experimental (black) Raman spectrum of Fe_2_[C_4_O_10_] at 65 GPa and calculated one based on Zn substitution for isostructural hypothetical Zn_2_[C_4_O_10_] (blue). Characteristic modes for CO_2_-V are marked with red asterisks (*). Calculated Raman spectrum for Zn_2_[C_4_O_10_] also shows a striking resemblance to that of Cd_2_[C_4_O_10_]^25^ confirming that these compounds are structurally similar.

Density functional theory (DFT) calculations for iron compounds can be demanding due to partially filled 3d-orbitals and variable spin states^54,55^. We investigated the influence of an on-site Coulomb correction with the +U-approach but found the effect to be very small. To avoid high-demand computations with complex spin systems, we performed the calculations by substituting iron with zinc atoms in the crystal structure. Zinc is slightly heavier than iron, hence we expect a minor red-shift of Raman bands; however, its ionic radius $({r}_{i}({{{{\rm{Zn}}}}}_{{{{\rm{VI}}}}}^{2+})=0.74{{{\text{\AA }}}})$ is comparable to that of high-spin Fe^2+^ $({r}_{i}({{{{\rm{Fe}}}}}_{{{{\rm{VI}}}}}^{2+})=0.78{{{\text{\AA }}}})$^56^. Additionally, Zn^2+^ exhibits no spin alignment or spin transitions, as all 3d-oribtals are occupied. Therefore, Zn^2+^ can be considered as a geometric analog for Fe^2+^, making it a suitable substitute that could facilitate the performed calculations. As a result, the experimental Raman spectrum for ${{{{\rm{Fe}}}}}_{2}[{{{{\rm{C}}}}}_{4}{{{{\rm{O}}}}}_{10}]-{Fd}\bar{3}m$ at 65 GPa is in a good agreement with the calculated one for hypothetical ${{{{\rm{Zn}}}}}_{2}[{{{{\rm{C}}}}}_{4}{{{{\rm{O}}}}}_{10}]-{Fd}\bar{3}m$ at the same pressure (Fig. 6), which is consistent with the assumption that there is essentially no coupling of the spin arrangement with the lattice dynamics and confirming the structural model. We have also measured and calculated Raman spectra for the coexisting CO_2_-V phase at 65 GPa and used it as a benchmark.

Three new modes at 692, 876 and 1068 cm^−1^ were observed in the Raman spectrum (Fig. 6). According to group theory analysis, the crystal structure of Fe_2_[C_4_O_10_] has following Raman-active modes: 3A_1g_ + 3E_g_ + 7T_2g_. We could assign the observed modes as T_2g_ (692 cm^−1^) and A_1g_ (876, 1068 cm^−1^) corresponding to complex vibrations of C-O bonds in [C_4_O_10_]^4-^ pyramidal anions, according to the DFT calculations.

A comparison of the experimental and DFT-calculated spectra for Fe_2_[C_4_O_10_] and Zn_2_[C_4_O_10_], respectively, demonstrates the expected consistency, even considering the complete substitution of iron with zinc in the crystal structure. Moreover, the calculated Raman spectrum for Cd_2_C_4_O_10_^25^ is also similar to that described above in terms of relative mode intensities and positions (Fig. 6).

### Compressional behavior of Fe_2_[C_4_O_10_]

The compressibility of Fe_2_[C_4_O_10_] was assessed by analyzing the unit cell volume data obtained from the solution and refinement of the crystal structure as a function of pressure^56^, in our case - in a pressure range from 65 to 25 GPa. Additionally, we performed DFT-based geometrical optimizations (= relaxation) of Fe_2_[C_4_O_10_] structures over a wider pressure range (0–90 GPa) to compare with the available experimental data. For the equation of state, the results of those calculations in which all Fe^2+^ ions were in the high spin state were employed, as these were more stable than low-spin configurations up to pressures of 95 GPa.

**Fig. 7:**
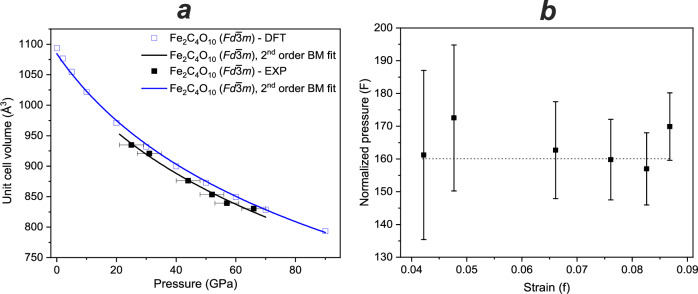
Evolution of the unit cell volume for Fe_2_[C_4_O_10_] on decompression. **a** Experimental and calculated unit cell volumes of Fe_2_[C_4_O_10_] as a function of pressure (filled and empty squares, respectively). The error bars for the volumes are smaller than symbols. The second-order Birch-Murnaghan equations of state for the calculated and experimental datasets are shown by blue and black lines, respectively; (**b**) normalized pressure (*F*) as a function of Eulerian strain (*f*) calculated for the *P*-*V* data collected on decompression; the horizontal dash line is the second-order fit of Birch Murnaghan equation of state.

Due to a limited number of collected pressure points and absence of an experimental *V*_0_ value (Fig. 3c), we found it reasonable to fit our P-V data using the second-order Birch-Murnaghan equation of state^57^ (Fig. 7a), with *V*_0_ treated as a fitted parameter. For the comparison with the computed data, they were fitted using the same approach. The resulting values for bulk modulus and unit cell volume extrapolated to zero pressure were similar (Table 2). Additional verification for the choice of the equation of state (EOS) comes from the plot of normalized stress *F* versus Eulerian strain *f*^58^ (Fig. 7b). This plot shows that the strain-stress data points, within the experimental uncertainties, follow the straight horizontal line, supporting the validity of the second-order Birch-Murnaghan EOS in describing the compressibility data.

**Table 2:** Comparison of bulk moduli for M_2_[C_4_O_10_] (M = Ca, Cd, Fe) as a function of ionic radius

Composition	Space group	Data		V_0_, Å^3^	K_0_, GPa	K’_0_, GPa^−1^	Ionic radius, Å^a^	Ref.
Fe_2_[C_4_O_10_]	${{{\rm{Fd}}}}\bar{3}{{{\rm{m}}}}$	DFT	2^nd^	1084(4)	152(3)	4.0	0.78	This work
			3^rd^	1092(2)	127(3)	4.93(12)		
		EXP	2^nd^	1059(17)	160(18)	4.0		
Cd_2_[C_4_O_10_]	${{{\rm{Fd}}}}\bar{3}{{{\rm{m}}}}$	DFT	2^nd^	1186.4(6)	132(3)	4.0	0.95	^25^
			3^rd^	1196.8(8)	112.2(5)	5.21(3)		
Ca_2_[C_4_O_10_]	${{{\rm{I}}}}\bar{4}2{{{\rm{d}}}}$	DFT	2^nd^	611(3)	110(3)	4.0	1	^26^
			3^rd^	616.7(2)	93.4(4)	4.83(2)		

^a^The values for ionic radii are based on^60^.

Nevertheless, the experimental data within the 25–65 GPa pressure range show a reasonable agreement with theoretical data obtained from DFT calculations (Table 2). Structural relaxation for the phase at different pressures revealed only minor differences between C-O and Fe-O bonds (Fig. 5) from experimental and calculated data. Such changes can directly influence the unit cell parameters and volume, which is obviously anticipated. Here, the discrepancy between experimental and theoretical unit cell volumes of Fe_2_[C_4_O_10_] does not exceed 3%, pointing to an accuracy of the structural model. Slightly higher values for the calculated unit cell volumes can be explained by the underbonding effect, where the values of bond lengths are slightly overestimated due to intrinsic short-comings of the DFT-PBE-GGA calculation approach employed here^59^.

The theoretical high-pressure data for similar Ca_2_[C_4_O_10_] and Cd_2_[C_4_O_10_] were also analyzed here in order to reveal the effect of cation radius on the bulk modulus within this group of carbonates. The calculated datasets for these compounds were fitted using both second-order and third-order Birch-Murnaghan equations. In our considerations we used ionic radii for six-fold coordinated atoms, and in the case of iron—in a high-spin state^60^. The summarized data for different carbonates containing [C_4_O_10_]^4-^ units is shown on Table 2. The bulk moduli for carbonates containing [C_4_O_10_]^4-^ units follow the trend of increasing bulk modulus with decreasing cation radius. While this behavior was previously demonstrated for calcite-type carbonates^61^, it is shown here for the first time in high-pressure carbonates with polymerized units. It is reasonable to expect that future synthesized carbonates with similar anions would follow the trend, highlighting the need for further investigations.

Among the iron carbonates, the compressibility and equations of state have been only analyzed for siderite Fe[CO_3_] and Fe_2_[CO_3_]_3_. It was shown that siderite exhibits an anisotropic compression behavior due to its trigonal crystal structure. The spin crossover in siderite at 44–45 GPa is accompanied by a sharp decrease of the unit cell volume from ~230 Å^3^ (high-spin Fe[CO_3_]) to ~ 208 Å^3^ (low-spin Fe[CO_3_]). There is also a significant increase of the bulk modulus from 110(2) GPa (*K’*_0_ = 4.6(2)) to 148(12) GPa (*K’*_0_ fixed at 5) and, consequently, in the acoustic velocity after the spin pairing is complete^48–50^. No phase and electronic transitions were identified in Fe_2_[CO_3_]_3_ on compression up to 40 GPa. The bulk modulus *K*_0_ derived from high-pressure XRD data is 138(34) GPa (*K’*_0_ fixed at 4)^62^. In contrast to FeCO_3_ and Fe_2_[CO_3_]_3_, Fe_2_[C_4_O_10_] contains different anionic unit, making a direct comparison of its bulk modulus and associated structural changes with those for other iron carbonates unreasonable.

### Implications to studies of high-pressure carbonates

Iron-bearing carbonates are well studied due to their significant role in deep Earth’s processes, for example, formation of carbonatitic fluids and their transportation to the surface via upcoming mantle flows (plumes)^39,40^. It was previously shown that the phase with a Fe_4_[C_3_O_12_] composition can be synthesized over a wide range of PT-conditions from different iron-bearing precursors such as FeO, Fe_2_O_3_ and FeOOH^41^; however, reliable structural data for this compound were initially unavailable. Later studies determined the trigonal crystal structure of Fe_4_[C_3_O_12_] and confirmed its stability above 74(1) GPa and 1750(100) K. In addition, the crystal structure of co-existing iron carbonate Fe_4_[C_4_O_13_] was also determined^17,63^. Our findings, based on the PT-conditions used for the synthesis of Fe_2_[C_4_O_10_], also suggest that Fe_2_[C_4_O_10_] could be stable under the conditions corresponding to the middle part of the lower mantle. However, the temperature used in the experiment (~3000 K) exceeds the one expected at the Earth’s depth corresponding to 65 GPa (~2400–2500 K)^64^. Given the significant temperature gradients inside the sample chamber after the second heating together with experimental temperature uncertainties, we propose that localized thermal anomalies, such as those around mantle plumes, could create short-lived higher-temperature conditions (at least 200–400 K above the surrounding mantle)^65–68^, where [C_4_O_10_]^4-^ units could be stabilized. Such plumes could also be enriched in carbon-bearing phases^69^ and hence provide a potential source of carbon for the synthesis. Consequently, the co-presence of high-pressure conditions, elevated localized temperatures, and carbon-rich environment within these thermal anomalies could create favorable conditions for the formation and stabilization of Fe_2_[C_4_O_10_].

## Conclusions

We report the successful discovery of Fe_2_[C_4_O_10_], a novel iron *sp*^3^-carbonate with [C_4_O_10_]^4-^ pyramidal groups, synthesized in diamond anvil cells under the high PT-conditions (65(4) GPa, 3000 (500) K), simulating carbon-rich environments probably existing in localized thermal anomalies in the lower mantle. The crystal structure of the compound has been determined by single-crystal X-ray diffraction, and it is similar to those of Mn_2_[C_4_O_10_], Cd_2_[C_4_O_10_] and Ca_2_[C_4_O_10_]. These compounds, together with hydrated barium carbonate, collectively constitute a group of highly polymerized carbonates. Fe_2_[C_4_O_10_] shows no phase transitions within a relatively broad pressure range between 65 to 25 GPa on cold decompression. The structural and spectroscopic characteristics of Fe_2_[C_4_O_10_] were examined in conjunction with DFT calculations, confirming the accuracy of the structural model for Fe_2_[C_4_O_10_] at 65 GPa and during decompression. Additionally, DFT calculations suggest a high-spin/low-spin transformation of Fe^2+^ atoms at 95 GPa.

## Methods

### Preparation of diamond anvil cell

We used DAC with the mini-BX90 design^70^ to generate high pressure in the experiment. Diamond anvils with culet size of 200 µm and an opening angle of ±30° were fixed in WC seats and aligned to ensure the uniaxial compression behavior. A rhenium gasket with ~200 µm thickness was placed between diamond anvils and was pre-indented to a thickness of approximately 30 µm. A circular hole with a diameter of approximately 100 µm was drilled by a Nd:YAG pulsed laser in the center of the indentation to prepare a sample chamber.

The single crystals of ^57^Fe-enriched hematite (α-^57^Fe_2_O_3_) were grown at 7 GPa and 1073 K in a 1200-tonne Sumitomo press installed at the Bavarian Geoinstitute^45^. As a precursor, a 1:1 mixture of a powder of non-enriched hematite (α-Fe_2_O_3_) of 99.998% purity and a pure powder of ^57^Fe_2_O_3_ (96.64%-enriched) was used. A particle of dark-brown α-^57^Fe_2_O_3_ with dimensions of 40 × 30 × 5 µm was placed inside the sample chamber (Fig. 1a). Then, the DAC was cooled down to approximately 100 K, and cryogenically loaded with CO_2_-I (dry ice)^32^. The loading process was carried out under an inert argon atmosphere to minimize contamination from atmospheric moisture. In the experiment, CO_2_ served as both a pressure-transmitting medium and a chemical reactant^71^. Carbon dioxide is a non-hydrostatic pressure-transmitting medium, and based on previous similar studies^26,28–30^, we estimate the uncertainty in the pressure determination to be around 4 GPa. Experimental pressures in the sample chamber were determined from the positions of XRD lines of CO_2_-V^43,44^. Due to non-hydrostatic effects, the pressure was unevenly distributed across the sample chamber, therefore we determined the pressures at the specific positions where the grains of Fe_2_[C_4_O_10_] were located.

### Laser heating

The DAC compressed to 65 GPa was laser-heated twice. The first heating was performed using an in-house double-sided laser heating system, equipped with a pulsed CO_2_ laser (Coherent Diamond K-250, λ = 10.6 µm)^72^. The sample was heated for 5 min with a maximum temperature of ~2000 (±100) K. The temperature was controlled by adjusting the radiation power from both sides of the sample. The laser was focused on a ~ 30 × 30 µm area, thereby providing uniform heating of the whole sample (Fig. 1a).

The second heating procedure was conducted using a one-sided laser-heating system at the ID27 high-pressure beamline (Nd:YAG IR laser, IPG Photonics, λ = 1064 nm, ESRF, Grenoble, France). The laser beam was focused on the central part of the sample’s surface, forming a spot of approximately 10 × 10 µm. The sample was heated up to a maximum temperature of ~3000 (±500) K for a few seconds (Fig. 2a). Due to the localized nature of the heating, we expect that the temperature gradients across the sample could reach ±500 K.

### X-Ray diffraction experiments and data processing

Each laser heating treatment described above was followed by a detailed X-ray diffraction mapping in order to determine the best spots for collecting of single-crystal X-ray diffraction data. Two-dimensional X-ray diffraction maps were collected from the entire sample chamber covering the sample, pressure-transmitting medium and partially rhenium gasket. The precision of the collected maps is defined by the scan ranges along the *y* and *z* axes and the number of steps (frames) per line. XRD maps were analyzed with the XDI software^73^; the DIOPTAS^74^ software package was used for phase analysis. The calibration parameters for XDI and DIOPTAS (sample-to-detector distance, coordinates of the beam center, tilt angle, and tilt plane rotation angle of the detector images) were refined using powder XRD pattern collected from NIST CeO_2_ standard.

X-ray diffraction data collection at 65(4) GPa was performed at ID27 high-pressure beamline at ESRF, Grenoble, France (λ ~ 0.37 Å, EIGER2 X CdTe 9 M photon-counting detector, X-ray beam size ~ 1.5(*H*) x 1.5 (*V*) µm^2^). The single-crystal XRD images were collected while rotating the DAC about a single *ω*-axis from −30° to +30° with steps of 0.5° and 1 s acquisition per frame.

The DAC then was decompressed in five steps down to ~25(4) GPa and the XRD data were collected at ID15b high-pressure beamline, ESRF (λ ~ 0.41 Å, EIGER2 X CdTe 9 M photon-counting detector, X-ray beam size ~ 1.5(*H*) x 1.5 (*V*) µm^2^). Similar to the experiment at ID27, the XRD maps were used to identify the best spots for SCXRD data collection. The pressure on decompression was estimated using a diamond anvil Raman gauge^75^. Below 25 GPa, the sample became amorphous and no reasonable XRD data could be collected.

After both laser heating treatments, the reaction products were composed of multiple crystalline samples. These samples consist of randomly-oriented crystalline grains with varying sizes, resulting in a typical 2D XRD picture, where smaller grains produce powder rings, and larger grains appear as distinct spots. The DAFi^76^ software, dedicated for handling multi-grain XRD data, was used to identify the most-intense crystals suitable for further processing in Crysalis^Pro^ (unit cell determination, integration of the reflection intensities and empirical absorption correction). The calibration of the instrument model of CrysAlis^Pro^ was performed using a single-crystalline vanadinite Pb_5_(VO_4_)_3_Cl (*a* = 10.3174 Å, *c* = 7.3378 Å, *Z* = 2, space group *P*6_3_/*m*). The calibration parameters included the sample-to-detector distance, the detector’s origin, offsets of the goniometer angles, rotation of the X-ray beam and the detector around the instrumental axis.

The obtained data on unit cell volumes as a function of pressure were processed using EOSFIT7-GUI software^57^. The *P*-*V* dataset was fitted using the third-order Birch-Murnaghan equation of state^56^ to derive the values for bulk modulus *K*_0_, the unit cell volume extrapolated to zero pressure *V*_0_ and bulk modulus derivative *K’*_0_. Birch-Murnaghan equations of state are widely employed for studies of carbonates at high pressures, as well as carbonates with similar anionic groups^25,26^, therefore this approach was also adopted in the current work.

### Structure solution and refinement

The crystal structures of iron oxides and carbonates obtained in this study were solved using SHELXT^77^, a structure solution program that uses the algorithm of intrinsic phasing. After structure solution positions of iron atoms were determined, while the remaining atoms (carbon and oxygens) were located from the difference Fourier maps. The crystal structures were refined against *F*^2^ on all data by full-matrix least-squares with the SHELXL^78^ software. SHELXT and SHELXL programs were implemented in the Olex^2^ software package^79^. The detailed summary of the crystal structure refinements at different pressures together with unit cell parameters, atomic coordinates, and atomic thermal displacement parameters is given in Table 1.

### Raman spectroscopy

The Raman map with dimensions 60 × 70 µm^2^ was recorded from the DAC at 65 GPa heated to ~3000 (±500) K. The data were collected using a dedicated setup comprised of WITec UHTS300 spectrometer (spectral resolution 0.1 cm^−1^) with a motorized stage alpha300R confocal microscope and a DR316B low dark-current deep depletion CCD camera. Raman spectra were collected using a 532-nm excitation UHTS300S_GREEN_NIR laser source and 100 mW of its power with a 50x/0.35 Olympus SLMPL objective. The map obtained is based on 16800 Raman spectra (120 points for each of 140 map lines with 1 s integration time) collected from studied rectangular area.

### Density function theory calculations

First-principles calculations were carried out within the framework of density functional theory (DFT), employing the Perdew-Burke-Ernzerhof (PBE) exchange-correlation functional and the plane wave/pseudopotential approach implemented in the CASTEP simulation package^59,80,81^. “On the fly” norm-conserving or ultrasoft pseudopotentials generated using the descriptors in the CASTEP data base were employed in conjunction with plane waves up to a kinetic energy cutoff of 1440 eV or 630 eV, for norm-conserving and ultrasoft pseudopotentials, respectively. The accuracy of the pseudopotentials is well established^82^. Spin polarised calculations were carried out both with and without a local Coulomb correction (+U). The CASTEP implementation of DFT + U adopts a simplified, rotationally invariant approach, where only external parameter required is the effective value of the on-site Coulomb parameter, U, for each affected orbital. In the present case, a value of 2.5 eV was chosen for the Fe-*d*-orbitals. A Monkhorst-Pack grid was used for Brillouin zone integrations^83^. We used a distance between grid points of <0.023 Å^−1^. Convergence criteria for geometry optimization included an energy change of <5 × 10^−6^ eV atom−1 between steps, a maximal force of <0.008 eV Å^−1^ and a maximal component of the stress tensor <0.02 GPa. Phonon frequencies were obtained from density functional perturbation theory (DFPT) calculations^84,85^. Raman intensities were computed using DFPT with the “2n + 1” theorem approach^86^.

## Supplementary information

Peer Review file

Description of Additional Supplementary Files

Supplementary Data 1

Supplementary Data 2

Supplementary Data 3

Supplementary Data 4

Supplementary Data 5

Supplementary Data 6

## Data Availability

The crystallography information files (CIFs) for the compound Fe_2_[C_4_O_10_] at different pressures reported in this study have been deposited at Cambridge Crystallography Data Center under deposition numbers 2393311-2393316. The data can be downloaded free of charge from https://www.ccdc.cam.ac.uk/data_request/cif.
