# Supplementary material for: High-pressure synthesis and crystal structure of iron sp3-carbonate (Fe2[C4O10]) featuring pyramidal [C4O10]4- anions
Source: Commun Chem. 2025 Mar 5;8:66. doi: 10.1038/s42004-025-01450-0 (PMC11883023; doi:10.1038/s42004-025-01450-0)
Supplement: Supplementary file 2 — Description of Additional Supplementary Files [file 42004_2025_1450_MOESM2_ESM.pdf]

Description of Additional Supplementary Files

File name: Supplementary Data 1

Description: Crystal data for $Fe_2[C_4O_{10}]$ at 65 GPa.

____

File name: Supplementary Data 2

Description: Crystal data for $Fe_2[C_4O_{10}]$ at 57 GPa.

____

File name: Supplementary Data 3

Description: Crystal data for $Fe_2[C_4O_{10}]$ at 52 GPa.

____

File name: Supplementary Data 4

Description: Crystal data for $Fe_2[C_4O_{10}]$ at 44 GPa.

____

File name: Supplementary Data 5

Description: Crystal data for $Fe_2[C_4O_{10}]$ at 31 GPa.

____

File name: Supplementary Data 6

Description: Crystal data for $Fe_2[C_4O_{10}]$ at 25 GPa.
